# The Bridge of Disconnect: An Angiographic and Cadaveric Study of Myocardial Bridges and an Overview of Its Embryology

**DOI:** 10.7759/cureus.97115

**Published:** 2025-11-17

**Authors:** Joshila Shibu, Susan Joseph, Tintu T Sukumaran, Rathi Sudhakaran, Saritha Sekhar, Asha Gopalakrishnan, Asha Joselet Mathew

**Affiliations:** 1 Anatomy, Amrita School of Medicine, Amrita Institute of Medical Sciences and Research Centre, Amrita Vishwa Vidyapeetham, Kochi, IND; 2 Cardiology, Amrita Institute of Medical Sciences and Research Centre, Amrita Vishwa Vidyapeetham, Kochi, IND

**Keywords:** coronary artery, ischemia, left anterior descending, myocardial bridges, myocardial loops

## Abstract

The heart muscle fibres of invertebrates and Pisces are nourished by the blood within them. In all other species, coronary arteries (CA) are the vasa vasora of the heart, nourishing its musculature. Proper coronary circulation is therefore crucial for sustaining the body. The present study comprised two entities, radio angiography and cadaveric heart dissection. For the radio angiographic study, 3,467 coronary angiograms performed between January 2022 and March 2025 were retrieved from the archives of the Department of Cardiology, Amrita Institute of Medical Sciences, Kochi, India. In the cadaveric study, we used 70 hearts from the soft tissue receptacle of the Department of Anatomy, Amrita School of Medicine. Meticulous dissection was carried out on the CA, and the tunnels were verified independently by the authors working in groups to avoid subjective bias. A total of 46 (1.33%) myocardial bridges (MB) were identified in the radio angiographic study, and 21 (30%) in the dissection study. They were all in the left anterior descending artery. We have addressed the development of CA and explored possible mechanisms for the formation of MB.

## Introduction

The heart muscle fibres of invertebrates and Pisces are nourished by the blood within them. In all other species, coronary arteries (CA) are the vasa vasora of the heart, nourishing its musculature. Proper coronary circulation is therefore crucial for sustaining the body. There are three sinuses of Valsalva at the base of the ascending aorta. The right posterior is described as non-coronary as it does not give rise to a CA. The anterior (right coronary) aortic sinus gives rise to the right CA (RCA), and the left posterior (left coronary) aortic sinus gives rise to the left CA (LCA) [1]. The two vessels lie epicardially in the atrioventricular groove. The left CA further divides into the anterior interventricular or left anterior descending (LAD) and left circumflex (LCX) arteries.

The artery should ideally be epicardial in position. In the condition referred to as myocardial bridge (MB), the CA dips or tunnels into the myocardium for a variable length and then reappears epicardially. The overlying myocardium is referred to as MB. The first anatomical description was by Reyman in 1737 [2] and then by Black in 1805 [3]. Geiringer in 1951 did extensive work on post-mortem hearts and coined the term intramural artery to describe the tunnelled segment of the CA [4]. With the advent of coronary angiography in 1958, the first radiological description of MB was made by Porstmann and Iwig in 1960 [5].

 The clinical implication of MB cannot be underestimated. It may present with a diverse clinical spectrum of rhythm disorders, angina pectoris, and myocardial infarction, indicative of coronary insufficiency - especially under conditions of heightened cardiac demand. This variability in expression underscores the necessity for comprehensive diagnostic evaluation, particularly in patients presenting with persistent chest pain despite non-obstructive findings on coronary angiography. Affected patients may experience impaired quality of life and adverse cardiovascular outcomes. Elucidating the anatomical and pathophysiological mechanisms of MB is critical for optimal therapeutic or surgical management.

Disruption during angiogenesis can lead to congenital defects of the CA. The common anomalies include mispatterning, structural anomalies, and anomalous communication. Despite the prevalence and clinical relevance of CA anomalies, much is not known about the cellular and molecular basis of the mechanics of their development.

## Materials and methods

The present descriptive observational study comprised two entities: radio angiography and cadaveric heart dissection.

For the radio angiographic study, 3,467 coronary angiograms performed between January 2022 and January 2025 were retrieved from the archives of the Department of Cardiology, Amrita Institute of Medical Sciences, Kochi, India. Using a draw of lots, the anatomists were assigned in pairs to three groups. Each group examined all angiograms for the presence of MB. The observations noted were confirmed by the senior cardiologist.

In the cadaveric study, we used 70 hearts from the soft tissue receptacle of the Department of Anatomy, Amrita School of Medicine, Kochi. The hearts were divided among the three groups at a minimum of 23 numbers, and a common mandate for dissection was outlined. The visceral pericardium was carefully stripped to expose the underlying coronary vasculature. Each coronary artery was then traced from its origin, and the major branches were systematically identified. Epicardial fat occupying the coronary grooves was meticulously removed by blunt dissection to delineate the vessels. The presence of myocardial tunnels was initially verified independently by each member of the team, followed by cross verification within pairs of a group, and subsequently validated between groups. This helped maximize accuracy and minimize subjective bias. Institutional ethical committee permission was granted for both parts of the study.

Inclusion and Exclusion Criteria

Coronary angiograms of adult patients of both sexes who underwent diagnostic coronary angiography and were concluded as normal. Angiograms of patients with occlusion, bypass surgery, and artefacts were excluded. For the cadaveric study, hearts with all coronary arteries intact were included, while those with structural damage either to the heart itself or to its coronary vessels were excluded.

## Results

Radio Angiographic Study

**Figure 1:**
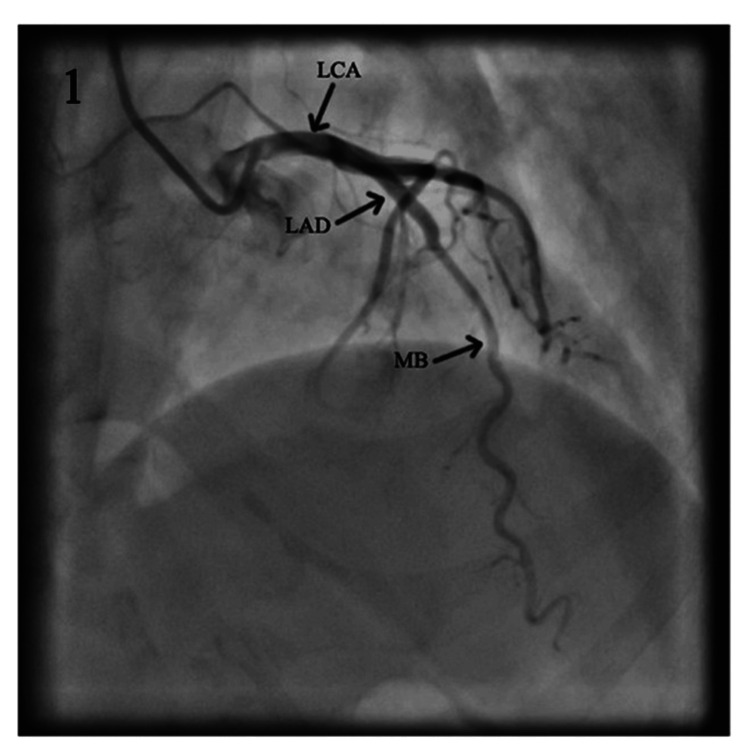
Coronary angiogram showing the left coronary artery and its branches; MB on the middle segment of the LAD. LCA: left coronary artery; LAD: left anterior descending; MB: myocardial bridge

**Figure 2:**
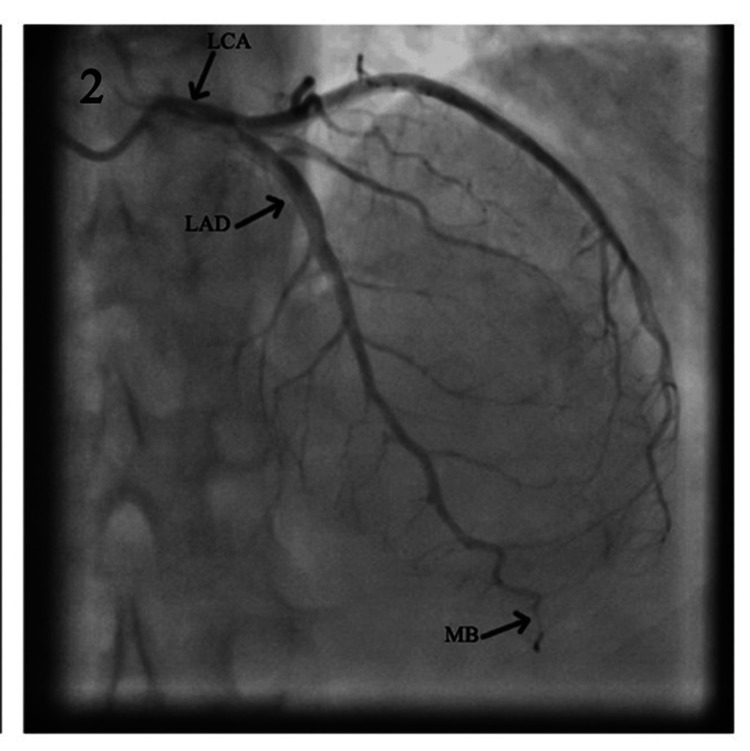
Coronary angiogram showing the left coronary artery and its branches; MB on the distal segment of the LAD. LCA: left coronary artery; LAD: left anterior descending; MB: myocardial bridge

A total of 46 (1.33%) MB were identified. They were all noted in the left anterior descending branch of the LCA, in two segments, as shown in Table 1 and Figures 1-2. Video 1 shows the coronary angiography illustrating a myocardial bridge on the midsegment of the LAD.

**Table 1:** Radiological findings of myocardial bridge (MB) on the left anterior descending artery (LAD).

Total number of angiogram studied	Total MB observed on LAD	Position of MB observed on LAD		
		Proximal LAD	Mid LAD	Distal LAD
3467	46 (1.33%)	-	45 (97.82%)	1 (2.17%)

**Video 1:** Coronary angiography illustrating a myocardial bridge on the midsegment of the LAD. A marked systolic reduction in luminal diameter is observed in the tunneled segment, followed by diastolic relaxation, reflecting the classical milking effect associated with myocardial bridging.

Cadaver Heart

**Figure 3:**
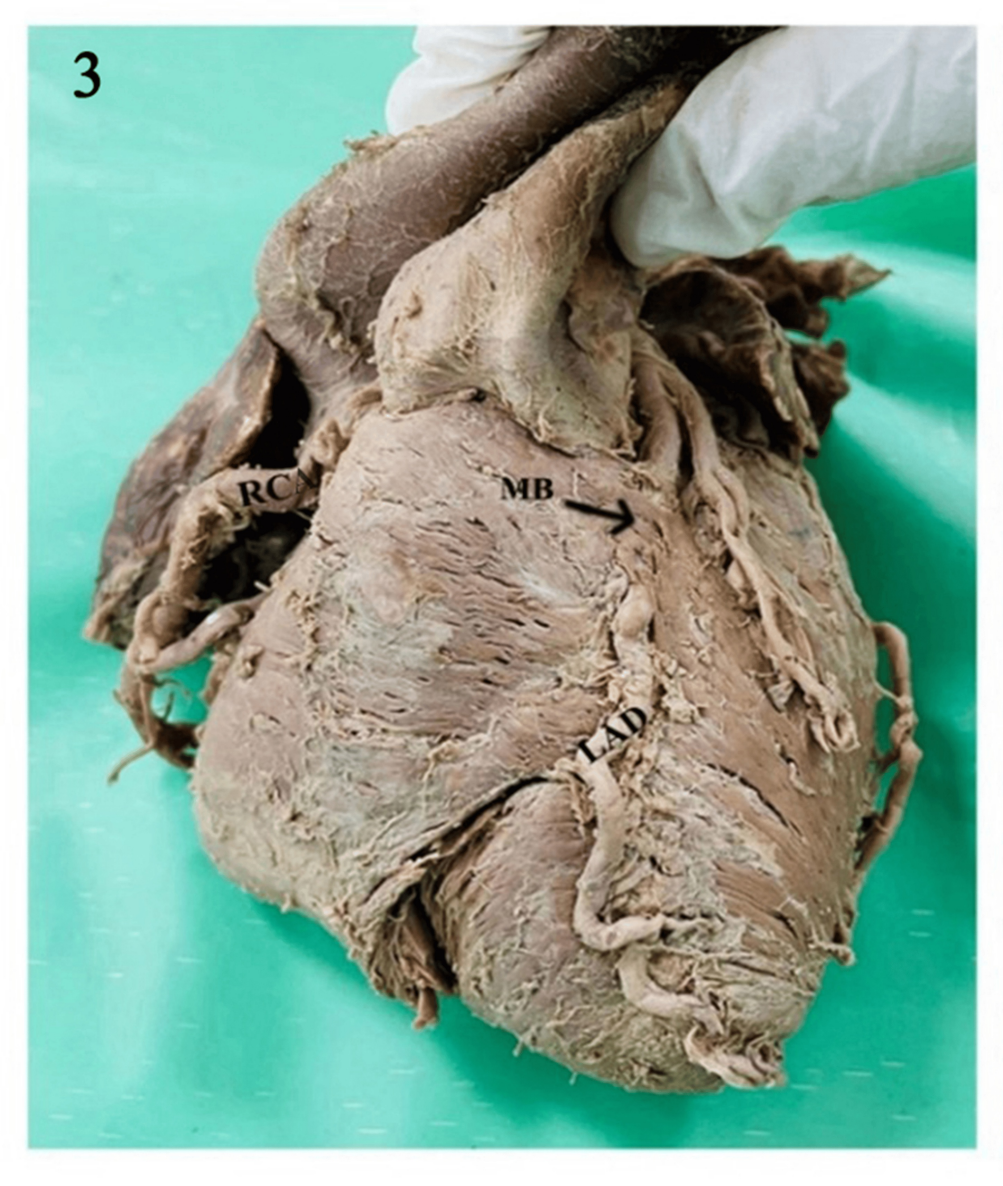
Cadaveric heart showing coronary arteries and their branches; MB on the proximal part of the middle segment of the LAD. LAD: left anterior descending; RCA: right coronary artery; MB: myocardial bridge

**Figure 4:**
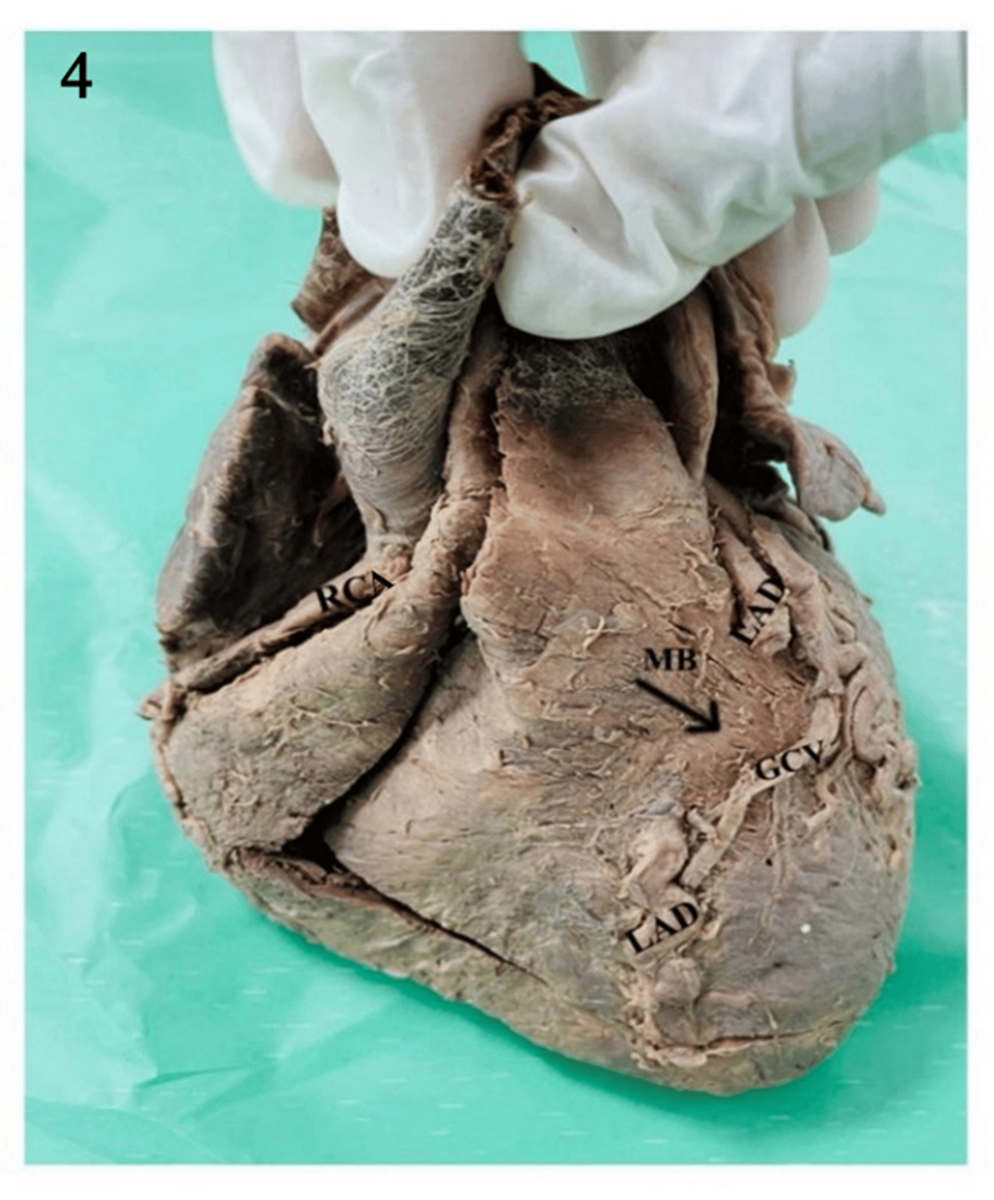
Cadaveric heart showing the coronary arteries and their branches; MB on the distal part of the middle segment of the LAD. LAD: left anterior descending; RCA: right coronary artery; MB: myocardial bridgeCV: great cardiac vein.

Out of the 70 hearts studied, MB were identified in 21 (30%) hearts. They were all noted in the middle segment of the left anterior descending branch of the LCA, as shown in Table 2 and Figures 3-4.

**Table 2:** Cadaveric findings of myocardial bridge (MB) on the left anterior descending artery (LAD).

Total number of heart studied	Total MB observed on LAD	Position of MB observed on LAD		
		Proximal LAD	Mid LAD	Distal LAD
70	21 (30%)	-	21 (100%)	-

## Discussion

The presence of MB is variable in its clinical presentation and can be asymptomatic or symptomatic. Some scientists consider them a benign anomaly with a good long-term prognosis [6]. An “anomaly” present in more than 1% of a large nonspecific population is considered a normal variant and not a true anomaly intrinsically capable of causing a disease state [7]. However, in many cases, MB is connected with heart rhythm disorder [8], angina pectoris [9], myocardial infarction [10], and sudden cardiac death [11], recognising it as an irrefutable cause of coronary insufficiency.

The tunnelling typically happens in the ventricular course. A subcategory of tunnelling that occurs in the musculature of the atrial wall, referred to as myocardial loop, has been described. The vessels involved in such an instance are the LCX and RCA. The clinical consequences of looping are said to be minimal due to the thinness of the atrial wall [12].

Variation in the frequency of myocardial bridging has been reported based on the modality of study. Although the rate of myocardial bridging is between 15% and 85% in autopsy studies, it is only seen in 0.5-2.5% of angiographic studies [13-15]. This finding compares well with that of the present study, wherein 46 (1.33%) angiograms showed bridging, and in the cadaveric study, 21 (30%) of the 70 hearts showed bridging. No myocardial loops were observed in our study. Coronary angiography is the prevailing technique for determining MB. The hallmark of bridging comprises a systolic tapering or “milking” of the artery with a characteristic “stepdown-stepup” sequence, delineating the affected area. The pretunnel area is almost always atherosclerotic [16].

Angiographic studies were carried on by several authors, such as Porstmann et al. [5] and Amplatz et al. [17]. Angelini et al. noted the disparity between the higher incidence reported from anatomical studies compared to angiographic studies [18]. The logical conclusion is that not all MB are symptomatic; when symptoms present, they can be attributed to ischemia [19].

Association of myocardial bridging with ischaemia and relief through surgical excision is well established [20]. A second way in which the presence of MB may predispose to coronary artery disease is through atherosclerosis. The intima of the vessel just under the MB is usually spared, but the prebridge segment is vulnerable to atherosclerotic patches [18]. Conversely, studies have proposed that MB offer shields to the artery from atherosclerosis, as opposed to non-bridged vessels [21].

Histological studies undertaken by Ferreira et al. showed that adipose and loose connective tissue separated the vessel adventitia from the muscle of the bridge [22]. They went on to classify the muscle bridge as superficial if the muscle bundles were oriented perpendicular or at an acute angle to the vessel and deep if the muscle bundles were longitudinally arranged and directed helically, transversely, or obliquely. They surmised that the helical fibers were more likely to distort the vessel and cause it to produce ischemia. Studies have shown that these muscular bands are present from birth [23].

The LAD is the most clinically important vessel that is bridged and usually sighted between its proximal third and middle third [24,25]. The middle portion of the LAD is listed as most frequently affected, though RCA and LCX may also be involved [26]. Loukas et al. in a study of 200 specimens reported the odds of succession of appearance of MB as on LAD, diagonal branch of the LCA, followed by the inferior interventricular branch of the RCA [27]. A panoptic study was carried out by Morales et al. [28] on 39 human hearts. All 39 (100%) showed MB over the LAD; additionally, 33% bridging was reported over the inferior interventricular branch and 18% in the first diagonal branch of LCA [28]. Among the 82 hearts studied by Baptista et al. [29], bridging over the LAD was reported to be 35.4%, while over the inferior interventricular branch, they reported 6.1% [29]. Authors such as Faruqi et al. [30], Channer et al. [31], and Irvin [32] concluded that myocardial bridging is generally confined to the mid LAD. In our study, all the bridges were noted in the LAD; 45 (97.82%) of the 46 were in the mid-segment of the LAD, keeping with results outlined in referenced studies; and one (2.17%) was on its distal part. In the cadaveric study, the artery involved in all cases was the mid LAD.

Among the therapeutic measures, β‐blockers and calcium channel blockers remain the mainstay of treatment in symptomatic subjects. If medical management is inadequate, supra-arterial myotomy is a viable option [33]. Potential complications of myotomy include wall perforation, ventricular aneurysm formation, and post-operative bleeding. While stenting can be explored as a recourse, it comes with drawbacks such as restenosis and stent fracture [34]. The major concern of CABG with regard to MB is graft failure [35].

Prior to 1989, it was postulated that coronary arteries and veins were outgrowths from the parent unit - the aorta and coronary sinus, respectively. Bogers et al., in their groundbreaking study, identified CA prior to the appearance of arterial orifices in the sinuses of Valsalva. This prompted a shift in the understanding of coronary arterial development, from the earlier notion of egression to the concept of ingression [36].

The proepicardium (PE) is an ephemeral extra-cardiac embryonic structure, which plays a crucial role in cardiac morphogenesis. In all vertebrates, the PE emanates from the lateral plate mesoderm. The mesothelial cells of the PE migrate towards the exposed myocardium as either clumps of cells or as a tissue bridge. They embrace the myocardium and form the third mantle of the heart, the epicardium [37].

**Figure 5:**
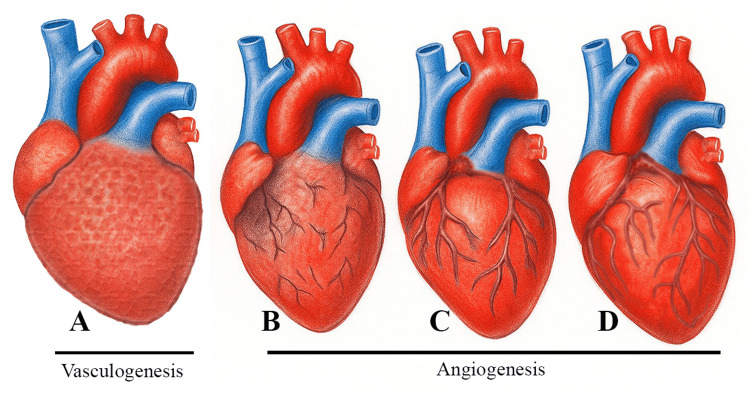
Schema of the development of the coronary vascular system. A - Vasculogenesis; B - Vascular plexus; C - Penetration of the aortic root; D - Two-vessel coronary arterial system Image Credit: Author Joshila Shibu

The intricate interlinking of epicardial and myocardial cells causes conversion of some of the epicardial cells into mesenchymal cells, which provides the antecedent cell lineage for coronary vessel development (Figure 5A) [38]. On the 25th day of intra-uterine life, haphazard angiogenic activity ensues in the myo-epicardial interphase. They subsequently abut on each other and amalgamate into a plexus, but unperfused (Figure 5B). When the vascular plexus ingresses towards and finally invades the aortic root, it is rapidly exposed to high systemic pressure, causing its perfusion (Figure 5C). Further remodelling and maturation occur through relocation and placement of smooth muscle cells, alongside a controlled burgeoning of some vessels and apoptosis of others (Figure 5D). Any irregularity during this exquisitely regulated activity can steer the way to congenital coronary artery anomalies. The embryological basis of MB is postulated as secondary structures formed by the migration of myocytes over the subepicardial coronary arteries [39]. Ramai et al. in 2018 reviewed and re-evaluated coronary artery development and advanced Boger’s theory of ingrowth of the coronary artery vasculature [40].

The principal limitation of the present angiographic study is the absence of evaluation of MB as a potential etiological factor for possible symptoms. Moreover, quantification of the morphological characteristics and dimensions of the MB would have provided a robust understanding of its contributions to symptom severity. In addition, the possibility of subjective bias in the designation of an arterial segment as bridged cannot be excluded. Finally, it must be acknowledged that coronary angiography possesses limited sensitivity for the detection of MB under resting conditions.

## Conclusions

An understanding of the location of the MB is important for the cardiologist and cardiothoracic surgeon while performing stenting or bypass for a good outcome. The study provides valuable prevalence and anatomical distribution data validated across imaging and dissection methods; further studies linking MB morphology with functional outcomes and refining diagnostic criteria are warranted. For the anatomy aficionado, its embryological basis is good grist for the analytical mill.
